# Implementing Complementary Approaches to Shape the Mechanism of α-Synuclein Oligomerization as a Model of Amyloid Aggregation

**DOI:** 10.3390/molecules27010088

**Published:** 2021-12-24

**Authors:** Marco Giampà, María J. Amundarain, Maria Georgina Herrera, Nicolò Tonali, Veronica I. Dodero

**Affiliations:** 1 Department of Clinical and Molecular Medicine, Norwegian University of Science and Technology, Olav Kyrres Gate 9, 7491 Trondheim, Norway; marco.giampa@ntnu.no; 2 Instituto de Física del Sur (IFISUR), Departamento de Física, Universidad Nacional del Sur (UNS), CONICET, Av. L. N. Alem 1253, Bahía Blanca B8000CPB, Argentina; mjamundarain@gmail.com; 3 Institute of Biochemistry and Pathobiochemistry, Ruhr University Bochum, 44801 Bochum, Germany; geor.herr@gmail.com; 4 BioCIS, CNRS, Faculté de Pharmacie, Université Paris-Saclay, 92290 Châtenay-Malabry, France; 5 Organic and Bioorganic Chemistry, Chemistry Department, Bielefeld University, Universitätstr. 25, 33615 Bielefeld, Germany

**Keywords:** protein aggregation, oligomer, α-synuclein, secondary structure, biophysics, model systems

## Abstract

The aggregation of proteins into amyloid fibers is linked to more than forty still incurable cellular and neurodegenerative diseases such as Parkinson’s disease (PD), multiple system atrophy, Alzheimer’s disease and type 2 diabetes, among others. The process of amyloid formation is a main feature of cell degeneration and disease pathogenesis. Despite being methodologically challenging, a complete understanding of the molecular mechanism of aggregation, especially in the early stages, is essential to find new biological targets for innovative therapies. Here, we reviewed selected examples on α-syn showing how complementary approaches, which employ different biophysical techniques and models, can better deal with a comprehensive study of amyloid aggregation. In addition to the monomer aggregation and conformational transition hypothesis, we reported new emerging theories regarding the self-aggregation of α-syn, such as the alpha-helix rich tetramer hypothesis, whose destabilization induce monomer aggregation; and the liquid-liquid phase separation hypothesis, which considers a phase separation of α-syn into liquid droplets as a primary event towards the evolution to aggregates. The final aim of this review is to show how multimodal methodologies provide a complete portrait of α-syn oligomerization and can be successfully extended to other protein aggregation diseases.

## 1. Introduction

In several neurodegenerative diseases, protein misfolding and aggregation are two phenomena that characterize the progressive course of the pathology [1,2]. Their occurrence often involves intrinsically disordered proteins (IDPs), which undergo a conformational change leading to misfolded conformation, which is responsible for the resulting protein aggregation [3]. All the steps of this complex mechanism involve heterogeneous and dynamic aggregated species, which continuously evolve in their structure, morphology, hydrophobicity, and solubility. This dynamism makes it difficult to characterize the entire aggregation process, and especially the species responsible for the toxicity. Several biophysical techniques such as spectroscopic and microscopic methods have been employed in recent years [4,5], to provide better characterization of the molecular nature of the oligomers, which are considered, from one side, the pathological culprits responsible for degeneration and, on the other, the most difficult species to investigate because of their structural heterogeneity, plasticity, and transitory nature [6]. Both soluble oligomers and insoluble fibrils have been related to tissue damage through the deterioration of surrounding cells in neurodegenerative diseases and systemic amyloidosis [7,8].

**Figure 1:**
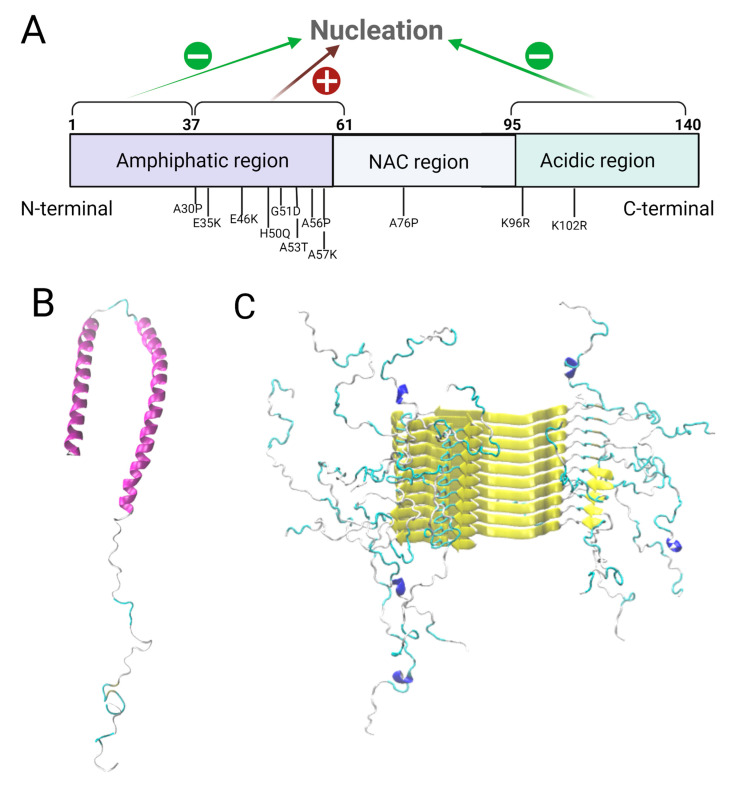
Structural properties of α-syn. (**A**) Schematic representation of the different protein domains: The amphipathic region which comprises the first 60 residues, the non-amyloid-β component (NAC) which is between residues 61–95 and the acidic region, located from the 96th residue to the C-terminal domain. These domains can have an activating (+) or inhibiting (−) effect on the nucleation of α-syn. Also here, we present different mutations related to the disease. (**B**) Structure of alpha synuclein monomer bound to a lipid micelle, obtained by nuclear magnetic resonance (NMR) (PDB ID: 1XQ8) [12] and (**C**) Atomistic detail of an alpha synuclein fibril obtained by solid state NMR (PDB ID: 2N0A) [16].

One of the most studied systems related to protein misfolding and neurodegenerative diseases is α-synuclein (α-syn), a 140-amino-acid protein present at presynaptic terminals both as soluble cytosolic and membrane-associated fractions. The accumulation of α-syn leads to the formation of Lewy bodies, which are composed of α-syn deposits and represent the pathological hallmarks of Parkinson’s disease (PD) [9,10] and multiple system atrophy [11]. α-syn can be divided into three different domains (Figure 1A): an N-terminal amphipathic region (residues 1–60) which presents five conserved lysine-rich repeats, the central non-amyloid-β component (NAC) domain (residues 61–95) which forms the core of α-syn amyloid fibrils, and the unstructured C-terminal acidic region (residues 96–140) [12]. While the first 37 residues of the N-terminal region and the C-terminal domain hinder nucleation, the second portion of the N-terminal domain favors it [13]. The structure of α-syn is highly dependent on environmental factors and the complexation state. For instance, helix-rich conformations are observed in micelles-bound α-syn (Figure 1B), while beta-sheets are predominant in fibril structures (Figure 1C) as described through nuclear magnetic resonance (NMR). Recently, the role of endogenous factors (lipids or proteins), exogenous factors (metals), and synthetic or natural inhibitors of the aggregation process were extensively reviewed [14,15] and are therefore not covered here.

**Figure 2:**
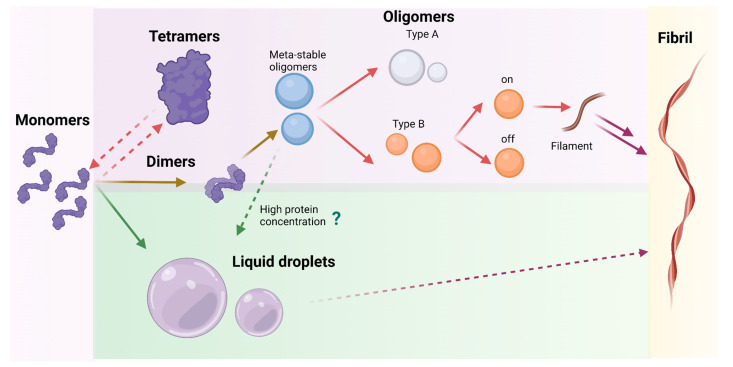
Illustration of the plausible mechanistic pathways of α-syn self-assembly process into fibrils. Monomers could self-assemble into dimers and then progressively into different oligomeric species (Meta-stable, Type A, Type B*_on_*, Type B*_off_*), some of which will form filaments and then the fibrils. Monomers can also form tetramers, which are considered non-aggregative species, that can dissociate again into monomers. Also, α-syn could undergo liquid-liquid phase separation, which could potentially undergo fibrillization. Created with Biorender.com.

The α-syn aggregation process is complex and heterogeneous, occurring through different early- and late-stage intermediates (Figure 2). Two main theories exist that explain the early stages of growth: the nucleation-polymerization and the nucleation-conversion-polymerization models. The first hypothesis is related to a conformational change of α-syn monomers from a disordered state into β-sheet structures; the self-association of small β-sheet multimers represents the rate-limiting step for the formation of the minimum template which initiates the aggregation process [17]. The second hypothesis assumes that the formation of disordered oligomers through intramolecular and intermolecular hydrophobic collapse of monomeric α-syn progressively evolves, at slow rates, into β-sheet oligomeric species [18]. Both theories agree with a necessary primary nucleation step for the progressive growth of early-stage species (meta-stable, type-A and type-B*_on_* oligomers) into protofibrils through monomer addition and, thereby, mature amyloid filaments. Recently, it has been observed that endogenous cellular α-syn exists largely as an α-helically folded, ∼58 kDa tetramer under native conditions and that the monomer represents a non-fully functional and less abundant species in normal cells. Given the much lower propensity of the native tetramer to aggregate into fibrils, it has been suggested that tetramers undergo destabilization of their helically folded conformation prior to α-syn aggregation into abnormal oligomeric and fibrillar assemblies [19,20] (Figure 2). Alternatively, α-syn can also aggregate through the formation of liquid droplets leading to phase separation and fibrillation [21]. Additionally, other oligomeric species that are not directly involved in fibrillation (type-B*_off_* oligomers) have been found. Thus, the identification and characterization of different α-syn early-stage aggregates represent a key point for obtaining structural information about the formation of mature α-syn fibrils and the wide variability of their polymorphs.

The understanding of which pathway characterizes the α-syn aggregation process in different conditions can be achieved only by investigating the mechanisms of formation from monomer to oligomers, tracing the morphological changes of α-syn over time. The combination of morphological and kinetic studies will allow identification of the most crucial steps of α-syn aggregation, especially early stages (Figure 2).

The aim of this review is to present recent advances regarding the description of the oligomerization mechanisms of α-syn proteins. We underline, showing some selected examples, how the combination of different experimental and theoretical approaches is necessary to clarify the mechanism of aggregation, the morphological evaluation of aggregates and the influence of early-stage species on the entire process in vitro and in vivo [22]. The selected experimental examples are based on the combination of spectroscopy, mass spectrometry or microscopy techniques. Additionally, since theoretical computational analysis is fundamental for rationalizing the data obtained by in vitro and in vivo assays, Monte Carlo (MC) and Molecular Dynamics (MD) simulations as well as Variational Bayesian Weighting results are presented because they contribute to the molecular understanding of both the kinetics and morphological properties of the amyloid aggregation process. This review has the general objective to offer an overview of the most recent discoveries on the aggregative process of α-syn made through the combination of various biophysical techniques in vitro or in vivo. A-syn is a suitable example of an amyloid protein for understanding the aggregation process, given its characteristic of aggregating in vitro more slowly than other amyloid proteins and the extensive conformational research performed on both soluble oligomers and fibrillar forms. The approach that will be highlighted in this review could interest a large audience of researchers who study the aggregation process of other amyloid proteins and who can therefore take inspiration from studies of α-syn to investigate other protein aggregation systems.

## 2. Mechanisms of α-Syn Aggregation In Vitro

### 2.1. Monomer Aggregation Propensity Linked to Conformational Transition

The heterogeneity of the oligomeric species has been assessed in several works with, often, apparently contradictory results, which can be attributed to different experimental conditions.

#### 2.1.1. Monomer Aggregation in the Nucleation-Polymerization Theory

In the nucleation-polymerization theory, the primary nucleation step is the first process that leads to the fibrillation of α-syn. Dimerization has been suggested as the earliest phase of nucleation of α-syn by which two monomers interact with each other in a specific conformation to form stable dimers. As the stability of dimers is directly correlated with their fluorescence lifetime when the protein is labelled with a fluorophore, a single-molecule fluorescence approach was employed to directly measure this property in α-syn dimers, by tethering unlabeled α-syn to a coverslip and by passing a solution of fluorescently labeled α-syn to form dimers [23]. This technique provides the possibility to explore which type of dimers the protein can adopt, how specific point mutations can affect the dimerization and therefore how the structural heterogeneity could lead to different aggregation pathways. Two classes of dimers with markedly different lifetimes were discovered. α-Syn mutations involved in the development of familial PD increase dimer stability, suggesting that the onset of the disease is defined by dimer formation, which is the very first aggregation event.

By assuming that the structural features of α-syn dimers are relevant to the aggregation pathway and the development of the disease, Churchill et al. described a diverse ensemble of dimers including elusive transient structured conformers. A collection of dimer structures using MC simulations with a ProFASI force field and implicit solvent has been created. The species with the highest content of secondary structure (not disordered) have been selected and used to perform MC constant-velocity pulling simulations. The force-extension curves were directly compared to measurements from single-molecule force spectroscopy. The discrete unfolding processes, which are characteristic of mechanically stable conformers, were evidenced through curves with steep variations (which the authors describe as rips). On the contrary, unstable structures presenting continuous unfolding processes were characterized by smoother curves that the authors describe as featureless. For all the assays, an α-syn dimer formed by two covalently linked monomers was used. Qualitatively, the in vitro and in silico force extension curves were similar, thus validating the computational models. From the in silico approach, the authors could correlate helix-rich dimers with continuous unfolding transitions, indicating that these are more unstable structures compared to dimers with a high content of β-sheets, which could be related to curves with discrete rupture events (a third group of the in vitro curves were consistent with the unfolding of stable structures). The regions more prone to form the dimer interfaces were identified as the N-terminus (1–20), the portion between the NAC and C-terminal regions (90–120) and the C-terminal domain (90–120). It was observed that most interfaces between monomers were disrupted in a single event taking place before the complete unfolding of the protein. Moreover, edge-to-edge interactions between β-strands were found responsible for dimer stabilization (and not the stacking of β-sheets). In this way, the combination of single molecule force spectroscopy and MC simulations allowed a molecular description of the structured transient dimers that might arise in the aggregation process, which include the more stable β-strand-rich dimers and the less stable dimers with a high content of α-helices.

By combining nano-electrospray ionization (N-ESI) mass spectrometry and ion mobility (IM-MS), it was observed that dimerization is favored under neutral pH conditions (around 7) due to a broad charge-state distribution, with nine or more negative charges linked with an extended conformation of the protein [24]. IM-MS analysis indicates that this protein is highly disordered. Phillips et al. described, through chemical crosslinking applied in conjunction with IM-MS, that the protein can adopt three distinct conformational families, Compact (~1200 Å^2^), Extended (~1500 Å^2^) and Unfolded (~2350 Å^2^) which correlate with those observed in solution. They emphasize the susceptibility of α-syn to environmental conditions, such as pH; the low sample pH, combined with the low pI of α-synuclein (4.67), results in less protein–protein repulsion and is therefore an environment conducive to oligomer formation. They could demonstrate that the species in the solution phase remain disordered overtime and that they adopt aggregation-related conformational modifications at the time of sequestration into oligomeric or fibrillar species [25]. Follmer et al., employing a similar approach, provided evidence that the disordered monomer exists in equilibrium with a dynamic dimer, which is prone to fibrillate [26].

**Figure 3:**
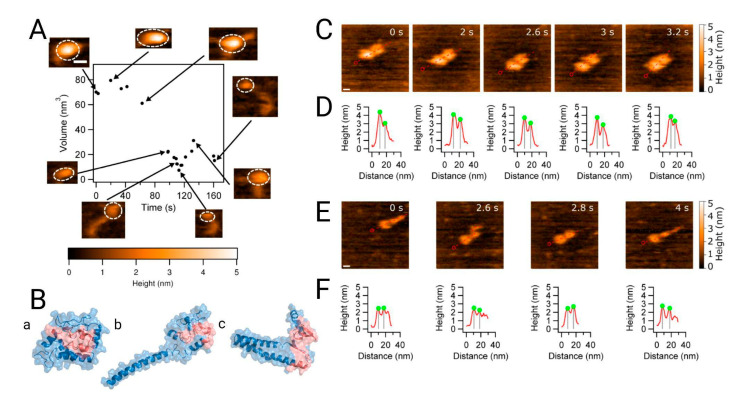
Analysis of the dynamics of alpha synuclein structure and morphology from the monomer to the dimer. (**A**) High-speed atomic force microscopy (HS-AFM) showing the monomer volume (black dots) with corresponding frames at specific time points. The insets in the figure, with dashed circles, indicate the segments used for volume analysis. (**B**) Discrete molecular dynamic simulation of the monomer. Structures from panels a to c represent the three most populated, lowest energy clusters, with populations of ~76%, ~14% and ~5%, respectively. The NAC region is shown in red. Morphology of dimers followed by HS-AFM z. (**C**) The α-syn dimer consists of two compact monomers that move apart and come together as the experiment progresses; scale bar is 5 nm. (**D**) Cross-sectional analysis of the dimers clearly shows monomer distance fluctuation. (**E**) Another type of dimer consisting of a globular monomer and an extended one. The scale bar is 5 nm. (**F**) Cross sections of (**E**) showing the effect of protrusion in the α-syn monomer. Extracted from [27].

In order to further characterize the structural dynamics of dimers and monomers, which are highly dependent on environmental conditions, the structural flexibility of α-syn monomers and dimers in phosphate-buffered saline buffer solution at pH 7.4 was assessed by single-molecule time-lapse high-speed atomic force microscopy (AFM). The results were then interpreted with the aid of replica exchange discrete molecular dynamics (REX/DMD) simulations [27]. For the computational models, the initial structures were obtained from unfolded conformations generated by a short high-temperature DMD simulation of the micelle-bound structure of α-syn (PDB ID: 2KKW) [28]; the dimer was constructed from two aligned monomers. The most populated clusters from the REX/DMD simulations were assessed and compared to the structures solved by AFM. Overall, monomers were found to be highly dynamic and assumed mostly compact, globular, helix-rich conformations (∼75% of the monomers), some of which contained protrusions (Figure 3A,B). These protrusions were also helical and some of them were linked to the rest of the protein through a short β-sheet. Protrusions in IDPs can have roles relevant to their function [29]. Globular monomers with fully elongated conformations, characteristic of IDPs, were also observed, but to a lesser extent. In contrast, dimers were less dynamic and the predominance of dimers formed by two compact monomers was observed (∼80% of the conformations) (Figure 3C–F). An alternative conformation composed of a globular monomer and an extended monomer was found in ~20% of the sampled dimers. The reduction in dynamic behavior can be attributed to the interactions that preserve the dimer as an entity. Within the dimers, monomers share a large hydrophobic interfacial surface formed mainly by residues in the NAC region which adopt β-sheet structures. Zhang et al. combined computational and experimental methods to explore the dynamic structural features of the flexible monomers and the more stable dimers, and showed that hydrophobic interactions in the NAC region are key to dimer formation.

#### 2.1.2. Monomer Aggregation in Nucleation-Conversion-Polymerization Theory

In the nucleation-conversion-polymerization model, the secondary structure modification of α-syn is considered the critical step in the formation of the nuclei responsible for the subsequent aggregation. A variety of complementary biophysical techniques have been employed to enable a structural and morphological characterization of how the conformational transition can impact the nucleation-dependent polymerization. For this purpose, α-syn aggregation was monitored by time-dependent circular dichroism (CD) experiments and Fourier transform infrared spectroscopy (FTIR), showing the secondary structure transitions overtime. Moreover, Thioflavin T (ThT) fluorescence was used to visualize the amyloid kinetic and formation of β-sheet-rich aggregates, and AFM was used to track the morphological transformation of these structures. All these techniques have been used for following the wild-type protein kinetic in comparison with the most relevant mutants of α-syn, to better characterize the process thanks to the subtle variations made by the point-mutation on aggregation behavior. Once the transition from unstructured conformations into β-sheet-rich fibrils passing through helix-rich intermediates was verified, the exploration of which segments of α-syn are critical for the formation of helical intermediates was performed by a Trp fluorescence study, together with multidimensional NMR analysis. These experiments evidenced the involvement of the N-terminal and central portions in the formation of helix structures and the mainly extended conformation of the C-terminus. Finally, the relevance of these helical intermediates in the pathological mechanism of α-syn were explored through other biological assays after isolation of helix-rich intermediates. The techniques that have been employed to demonstrate their role in toxicity include ANS fluorescence binding assay, cell viability assay (MTT) and cytometry analysis [30]. Several studies have suggested that α-syn can strongly bind to negatively charged membranes and adopt an α-helical structure [31,32]. This intermediate conformation has been hypothesized to enhance the aggregation ability of α-syn in the vicinity of membranes and thus the alteration of membrane integrity associated with α-syn pathogenesis [32]. Conversely, parallel beta-structures are not involved in cellular toxicity. A meticulous analysis based on far-UV CD, FTIR and fluorescence spectroscopy shows structural differences between the major subgroups of α-syn oligomers in combination with toxicity assays, finding that oligomers with parallel β-sheet architecture and with lower degrees of surface-exposed hydrophobicity are also formed in the early stages of the self-assembly process, but do not participate in toxicity. The parallel oligomers represent the aggregative form which is able to elongate rapidly and generate fibrils. In that case, rearrangement of the β-strands from an antiparallel to a parallel configuration seems to be the key step of these processes, which are required for the efficient elongation of these α-syn oligomers to generate the fibrils [33].

#### 2.1.3. The Conformational Dynamics of α-Syn Monomer Affect the Aggregation Process

The physical and structural properties of α-syn domains directly affect the morphology and kinetics of the aggregation process. Gallardo et al. performed deletion studies on α-syn to explore the role of the different α-syn regions and the A53T mutation in the nucleation and elongation of amyloid fibers. In order to do so, they applied CD and transmission electron microscopy (TEM) before and after the fibrillation of WT α-syn and its A53T mutant and used ThT fluorescence to assess the kinetics of nucleation and fibril elongation [13]. A quantitative approach by SDS-PAGE was employed to reveal the influence of the different α-syn regions on fibrillation, by measuring the percentage of initial monomers that become fibers. The nucleation time and elongation rate of truncated α-syn versions were measured by fitting the ThT fluorescence curves. Finally, the structural compatibility between fibers from different α-syn deletion mutants was assessed through cross-seeding experiments and TEM analyses. N-ter and C-ter truncated forms of α-syn showed a decrease in nucleation time compared to full length α-syn, implying that these regions inhibit nucleation and therefore amyloid aggregation, while the pre-NAC region (residues 31–62) was revealed to be a nucleation-promoting region involved in protofiber interactions. Two hypotheses have been suggested for the inhibition: the helical propensity of the N-ter region could impair the beta structure formation, or an interaction between N-ter and C-ter sequences could hide the NAC region, thus preventing aggregation. The results presented by Gallardo et al. indicate that the deletion of the C-ter region has a greater impact on fibrillation than deletion of the N-ter region due to its interaction with the central hydrophobic region. The N-ter/C-ter interaction, even if it takes place, is therefore not primordial to fibrillation inhibition. Furthermore, the deletion of the pre-NAC region produced fibers with an altered morphology. A53T hinders N-ter and C-ter mediated fibrillation inhibition, and so it stimulates nucleation and elongation of α-syn amyloid fibers. It decreases the nucleation time and increases the elongation speed. No differences were observed for the deletion of the initial portion of the N-terminal region and the C-terminus in this mutant. The seeds obtained from this mutation were more easily elongated (even by non-mutated monomers) than the WT seeds. Therefore, mutations in different domains of α-syn alter the aggregation kinetics, propensity and morphology of the fibers. While the WT pre-NAC region favors nucleation and fibrillation (Figure 1A), the C-terminal domain and the first N-termini residues inhibit these events.

At the current stage, it is well known that the highly dynamic conformation of the monomer, which is dependent on environmental conditions and complexation state, affects all types of aggregation pathways such as nucleation-polymerization or nucleation-conversion-polymerization. Therefore, the complete characterization of the structural ensemble of α-syn monomers at an atomic level is relevant since the different conformers available for the protein can be associated with particular functions. These are not only affected by environmental conditions (pH and temperature) but, more importantly, are completely linked to the complexation state, i.e., α-syn adopts different conformations as a monomer in solution, when it is bound to membranes, if it is part of dimers, small oligomers, or forming higher-order structures. Distance distributions obtained from time-resolved FRET were employed to design and refine potential functions used in discrete MD simulations [34]. From these experiment-guided simulations the conformational ensemble of α-syn was explored, and then clustered to obtain eight representative structures. The structural features of the conformers of each cluster were contrasted with the previous characterization of α-syn in different cellular contexts. In some cases, such as α-syn bound to a micelle membrane (PDB: 1XQ8) and as a part of fibrils (PDB: 6A6B), the root-mean square deviation was computed between the structures in each cluster and the experimentally-determined structures. Each representative structure could be associated with a preexisting intermediate related to at least one complex: dimer, tetramer, oligomer, membrane-bound and fibrils. The structural properties of the ensemble were validated with far-UV CD (secondary structure content), cross-linking mass spectroscopy (local proximity of different regions of the protein) and single-molecule protein-induced fluorescence enhancement. From this last assay, long transition times (of milliseconds) were observed for some species, indicating substantial local stability which could favor the association of this protein with other biomolecules. In sum, a set of in vitro techniques were used to guide and evaluate a DMD simulation performed to map the conformational landscape of the α-syn monomer. Representative structures related to specific interactions with other molecules were found; these conformations might define its function in normal cellular activity as well as in pathogenic cases due to aggregation [35].

#### 2.1.4. Meta-Stable α-Syn Oligomers (Meta-αS-Os)

Before the formation of stable oligomers and fibrils, α-syn can even form small meta-stable α-syn oligomers (Meta-αS-Os). These structures have been characterized using a combination of ThT fluorescence spectroscopy with TEM and static LS to show that these soluble species are spherically homogeneous. Successively, CD analysis and ATR-FTIR have been employed to confirm the mainly disordered/random structure of the assemblies. These techniques showed that they have a metastable nature with a secondary structure similar to the monomer and that they are sensitive to environmental stimuli. These Meta-αS-Os are capable of promoting the formation of on- and off- pathway oligomers and can be converted under external stimuli into temperature-sensitive self-associative oligomers or NaCl-induced non-fibrillating oligomers [36].

Many studies have shown a wide size heterogeneity of α-syn oligomers during their aggregation mechanism. A preliminary characterization of the ensemble of α-syn oligomers in terms of size and level of heterogeneity has been performed by HPLC-SEC (Size Exclusion Chromatography) to assess the final composition of the purified samples and by native PAGE gel to define the apparent molecular masses. The results have been successively implemented by analytical ultracentrifugation (AU) measurements of sedimentation velocities and CryoEM, which allowed us to define two major size subgroups of oligomer species: 10S and 15S oligomers subgroups. Their overall morphologies have been then characterized by means of AFM techniques, demonstrating sphere-like morphologies, and similarly in TEM images [33].

As mentioned before, α-syn oligomers can be classified into either on-pathway or off-pathway intermediates in the fibrillation process. Generally, type-A oligomers are precursors which do not have a defined structure and are most probably related to Meta-αS-Os. These structures can be converted into more compact type-B oligomers with amyloid-like β-sheet conformation which participate in fibril production. Among type-B oligomers, it is possible to distinguish between two types of oligomers: type-B*_on_* which play the most important role in the fibrillation process and have been conformationally studied through SAXS analysis [37], and type-B*_off_*, identified by CryoEM, which are instead characterized by a high level of antiparallel β-sheet content and whose transformation into parallel sheets is energetically unfavorable, such that they no longer participate in fibril formation [33,38].

In order to characterize the size and shape of type-B*_off_* oligomers and to understand if they form spontaneously or derive from the conversion of type-A oligomers, the structural information of Meta-αS-Os has been studied with small-angle neutron scattering (SANS) [39], which represents a promising technique suitable for defining protein structure existing in a transient and disordered state. SAXS cannot be employed in this specific case because meta-stable structures usually exhibit a low scattering intensity. The advantage of SANS is to increase the scattering intensity of disordered proteins by using deuterium oxide that can control the scattering contrast between the proteins and the solvent. In this case, the combination of biophysical tools came in handy for the quality characterization of the sample containing Meta-αS-Os, in order to be sure to perform SANS analysis with the type of aggregates of interest. These well-characterized Meta-αS-Os have been subjected to AFM under different stimuli and are proven to be able to convert into two different types of oligomeric species: self-associative type-B*_on_* oligomers and non-fibrillating type-B*_off_* oligomers.

The heterogeneity of structure and topology of oligomeric species of α-syn have also been analyzed by ESI-Ion mobility mass spectrometry, showing that dimers to hexamers often coexist with various morphologies, suggesting that self-assembly follows an isotropic growth pathway [40]. In detail, it was found that lower-order oligomers are structurally heterogeneous with unstructured assemblies. Instead, higher-order oligomers were shown to be compact with ring-like structures and this particular structure is crucial for inducing, through the cell membrane, Ca^+^ influx and neurotoxicity in neuroblastoma cells. Therefore, compact higher-order ring-like oligomers are associated with intracellular seeding.

More recently, by combining Small-Angle X-ray Scattering (SAXS) and Variational Bayesian Weightings, Moretti, et al. explored the species of α-syn oligomers present at the prefibrillar state. The most prevalent form of oligomers for WT α-syn were unfolded monomers and trimers, with higher helical content at low temperatures, and rich in β-strands at high temperatures. In contrast with previous works, the tetramer structure was not observed for the WT protein. The detection of the tetramers has been widely discussed and the reproducibility of the experiments has been questioned due to their dependence on physical and chemical conditions as well as on the biological source of the protein [41]. However, for the most pathogenic mutation, G51D, the most abundant species were β-strand-rich tetramers for all temperatures. The most prevalent form of A53T mutant was also the β-strand rich tetramer, but with a decrease in predominance in favor of monomeric forms at low temperatures (25 °C). Whether these tetramers trigger the nucleation of fibrils or represent the neurotoxic species has not been explored yet. This methodology also described well the decrease in oligomerization propensity of the E46K mutant, which favors interactions between the N- and C-termini, leading to a prevalence of monomers.

#### 2.1.5. High and Low FRET Oligomers Subtypes

One strategy which has been employed to distinguish monomers from oligomers is to covalently label each monomer either with the same fluorophore or with two different red and blue fluorophores. In the first case, the distinction can be based on fluorescence intensity or the number of photobleaching steps, while in the other oligomers, it can be detected by the presence of both fluorophores in the same spatial region, thus providing the possibility to perform intramolecular fluorescence resonance energy transfer (FRET) at the same time [42]. This latter approach, in particular, can be employed to recover information about the number and concentration of the aggregates in a sample, as well as the size and structure of oligomers. The approach has the limitation of the uncertainty of detecting oligomers with the same size and structure in any given study. Therefore, using high-resolution AFM together with single-molecule fluorescence represents a complementary strategy to more accurately measure aggregate size and follow dynamics [42]. By applying this method to study α-syn aggregation, it was possible to identify two distinct subtypes of α-syn oligomers: species with high and low FRET. By applying a global kinetic analysis, it has been shown that the α-syn aggregation is characterized firstly by transition of the initially formed oligomers (low FRET, globular) to more compact oligomers (high FRET) which successively proceed to fibril formation by monomer addition. It could be possible to determine, at the molecular-level, the concentration and numbers of aggregates necessary for the effective seeding of α-syn. It has been demonstrated that very high numbers of seeds are required to achieve efficient seeding, thus suggesting another possible mechanism for the prion-like spread of the disease. By combining the biophysical assay with reactive oxygen species measurements, it has been found that the high FRET species are significantly more effective at inducing production of reactive oxygen species in neuronal cells than the low FRET species, thus suggesting that templated seeding occurs together with oligomer-induced cellular stress [43]. Single-molecule FRET has been used to study the aggregation of pathological mutants of α-syn. An exhaustive comparison of both the aggregation kinetics and the structural properties of the ensemble of oligomers was generated. This comparison was useful to analyze the behavior of mutants with respect to the wild type and to draw considerations regarding the anomalous behavior of mutants that leads to higher incidence of the disease. Studying the behavior of mutated species with a single-molecule approach or other biophysical tools improves our understanding of the amyloidogenic behavior of the wild-type protein, since they are similar. By this strategy it was possible, for example, to demonstrate that the properties of the oligomer species produced during oligomerization might be more relevant than their absolute concentration for triggering neurodegeneration [44].

#### 2.1.6. Towards a Combination of All Types of Aggregation Models

Once the oligomers have formed, the next step towards fibrillation could be either interactions between oligomers, or the addition of monomers to the oligomers. In fact, by following the aggregation process of α-syn via dual color FCCS (fluorescence cross-correlation spectroscopy), in which two fluorophores are employed simultaneously on monomers and tandem-oligomers, it has been shown that, during the early stages of aggregation, the oligomer-oligomer interactions seem to drive the fibrillization more than other types of interactions between oligomers and monomers, suggesting that early-stage oligomers do not behave as seeds by the addition of monomers [45]. The advantages of using two fluorophores are: (i) ensuring that particles labeled with two colors represent aggregates, (ii) sensitive discrimination between small differences in molecular weight between two species, (iii) detection of aggregates formed from different proteins [46,47]. Moreover, this technique allows the study of the properties of oligomers of different sizes in a controlled way, using engineered oligomers which are obtained by connecting two, four, or eight monomers head-to-tail with a three-amino-acid linker between each repeated domain [48]. These engineered oligomers can be successively characterized by a standard FCS assay, thus providing insights relative to the hydrodynamic radius as a parameter of the degree of compactness and size which can be correlated to values obtained by HPLC-SEC and DLS.

Recently, Silva et al. provided a framework to study the early stages of amyloid conversion through biochemical, kinetic, and structural studies combining ThT kinetic experiments, TEM proteolytic digestion, CD, ESI-MS, analytical size exclusion chromatography (A-Sec) and CryoEM [18]. Through this multidimensional approach, these authors compared wild-type α-synuclein and the A53T mutant (point mutation bearded by PD cases), that led to the distinguishing of oligomer and protofibril interconversions and suggested a new model of aggregation, which reconciles the nucleation-polymerization and nucleation-conversion-polymerization models. In this work, amyloid formation is not considered as a process in which species are formed sequentially. Rather, multiple species are thought to coexist at each moment of the kinetic in different proportions. While wild-type α-syn seems to preferentially follow a nucleation-conversion-polymerization model in which disordered oligomers are first converted to more organized β-sheet structures and then start to form mature fibrils, the A53T mutant has a constant pool of interconverting multimers and small oligomers that better fits the nucleation-polymerization model, in which structured species are rapidly formed in the early stages and are continuously accessible to incorporate monomers. This behavior reflects the exponential burst of the A53T kinetic profile during protofibril growth, with the absence of large oligomers (LgO) in comparison to wild-type α-syn, and the presence of small oligomers (SmO) whose amount remains constant, as confirmed by A-Sec. The sequestration of wild-type α-syn monomers as LgO probably limits the availability of conformational monomers that can be incorporated into the growing aggregates [18]. Furthermore, wild-type α-syn is dependent on a heterogeneous nucleation process at the polymer-water interface (lag phase due to nucleation of monomers on the fibril surface) which the A53T mutant is able to overcome for growth (no lag phase) [49]. All these findings allowed both the characterization of the features of A53T that may explain the early onset of familial Parkinson’s disease cases bearing this mutation, and the development of a new approach to study the amyloid mechanism, considering all types of models together with the new phase separation hypothesis.

#### 2.1.7. The Involvement of the Surface Hydrophobicity of Aggregates

**Figure 4:**
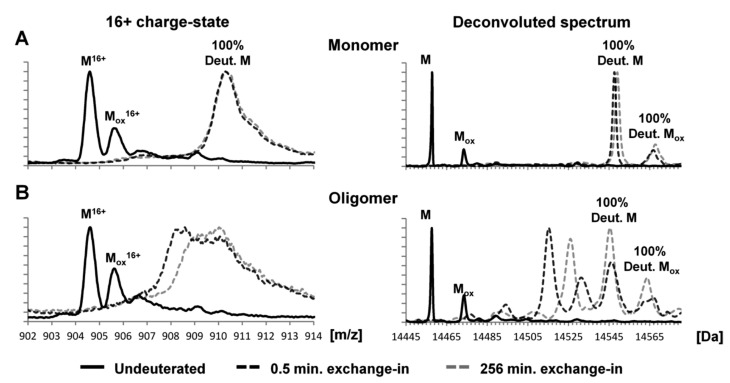
Global exchange profiles for α-syn monomers and oligomers comparing their non-deuterated state and deuterium incorporation after exchange for 0.5 and 256 min. Spectra represent the most abundant charge state (+16) and the deconvoluted spectrum. Peaks labeled M denote the molecular mass of the protein, while peaks labeled Mox denote oxidation products. (**A**) Deuterated monomeric αSN gave rise to a single unprotected population, exhibiting a mass increase of ~90 Da after exchange for 0.5 min. (**B**) Deuterated oligomeric αSN gave rise to two populations (i.e., protected αSN and unprotected αSN), with the unprotected population behaving like monomers and the protected population displaying a mass increase of ~58 Da after exchange for 0.5 min. Spectra were deconvoluted using MaxEnt 1. The raw mass spectra presented here were smoothed [52].

Another factor involved in the aggregation mechanisms is the surface hydrophobicity of oligomers and/or single protein aggregates. By implementing a single-molecule fluorescence imaging microscope with a blazed transmission diffraction grating placed before the image plane, it is possible to perform multi-dimensional super-resolution imaging to map the hydrophobicity surface of oligomers. For this purpose, specific single-molecule fluorescent emitters, such as the dye Nile Red (NR), are employed to emit a wavelength fluorescence emission that is highly dependent on the local hydrophobicity of its environment [50,51]. As a first attempt, the hydrophobicity of single protein aggregates is quantified by sPAINT (points accumulation for imaging in nanoscale topography). Since the surface hydrophobicity, or solubility of the protein aggregates, is thought to play a central role in the toxicity, sPAINT is a potential tool to probe sub-populations of oligomers/fibrils and to potentially correlate aggregate structure with hydrophobicity. Through this assay it is even possible to compare two different amyloid proteins and study how they behave differently in terms of aggregate distribution (morphology/size) and hydrophobicity [51]. The technique has been recently evaluated for its potentiality to map the surface hydrophobicity of α-syn oligomers at the nanoscale. From the raw data, it is possible to trace cumulative frequency histograms of mean wavelength of individual α-syn aggregates (hydrophobicity histograms), density plots of the mean wavelength and number of localizations of individual α-syn aggregates (hydrophobicity landscape), and finally density plots of the mean wavelength and hydrophobicity variability of individual α-syn aggregates (hydrophobicity heterogeneity). Spectral analysis of an entire kinetic of α-syn aggregation allowed the observation of changes in the surface hydrophobicity at the single-aggregate level, providing insights into how the surface properties and heterogeneity of aggregates change as aggregation proceeds [50]. The hydrophobicity of the oligomers was also investigated by Hydrogen-Deuterium Exchange Mass Spectrometry (HDX-MS), which permits evaluation of the solvent accessibility by hydrogen-deuterium exchange of the amide backbone [52]. These studies indicate similarities between oligomers and fibrils and highlight the presence of an N-terminal that is protected from isotope exchange. In addition, the HDX studies show that deuterated monomeric α-syn gave rise to a single unprotected population, exhibiting a mass increase of 90 Da after exchange for 0.5 min. On the other hand, the deuterated oligomeric α-syn gave rise to two populations (protected and unprotected α-syn), with unprotected population behaving as monomers and the protected ones showing a mass increase of 58 Da after 0.5 min exchange (Figure 4). Experiments with pulsed HDX, in which a variable narrow deuteration window is used to follow stable and metastable structure, shows that the aggregation of α-syn is driven by the hydrophobic NAC region [53].

Even though amyloid formation is driven mainly by the attraction of the hydrophobic cores, electrostatic interactions are also capable of modulating the nucleation and aggregation of α-synuclein. By a combination of in vitro and in silico experiments, Gaspar et al. showed that short-range attractive electrostatic interactions are key for nucleation and aggregation [54]. They combined ThT fluorescence assays at pH 5.5 of α-syn in the presence of α-syn seeds and anionic lipid membranes, and constant pH MC simulations of a coarse-grained model of α-syn over a layer of α-syn acidic C-termini. Secondary nucleation (the nucleation of new fibrils on the surface of existing fibrils) in pre-formed α-syn seeds and primary nucleation in anionic membranes were delayed and even impaired at high salt concentrations. While an increase in ionic strength screens the long-range repulsion between the anionic monomers and the negative catalytic surfaces, it also screens the stronger short-range attractive forces between the positively charged N-termini of the monomers and the negative counterparts (acidic C-termini and anionic lipid vesicles). The net effect is a reduction in attraction and impairment of aggregation.

Changes in the electrostatic properties of the protein have also been observed as a consequence of aggregation. In fact, a significant increase in pH has been observed during α-syn fibrillation in water [55]. Three different pH measurements, including solution state NMR spectroscopy, were conducted for two systems: WT α-syn and a mutant with 5 acidic residues changed into glutamines. Static light scattering and ThT fluorescence were additionally employed to verify that fibrillation was taking place. Far-UV CD Spectroscopy was also used to assess the transition from monomers (mostly random coil) to fibrils (high β-sheet content), and Cryo-TEM was employed to evaluate the morphology of the fibrils. Moreover, results were compared to constant pH Metropolis MC simulations on a model system based on the structure PDB (ID: 2N0A). Overall, Pálmadóttir et al. could conclude that the upshift in the pH value was caused by an uptake of protons in the acidic C-terminal domain during the fibrillation formation. As a consequence, the electrostatic repulsion between the acidic C-termini within the fibril is lowered, favoring aggregation.

### 2.2. Tetramers of α-Syn

In recent years, tetrameric αSyn has been proposed as a novel physiological state, with a strong ability to resist aggregation. Familial mutations seem to ablate tetramerization and reconfigure polymorphic fibrillization. The heterogeneity of the oligomeric species has been assessed in several works with, often, apparently contradictory results which can be attributed to different experimental conditions. From a combination of solution NMR, CD, EM, chemical cross-linking and mass spectrometry [56], it was shown that α-syn forms stable tetramers in the absence of lipid bilayers or micelles. The canonical mechanism by which the tetramer assembles remains elusive. Via a combination of solution NMR, CD, EM, chemical cross-linking and mass spectrometry [56], it was shown that α-syn forms stable tetramers in the absence of lipid bilayers or micelles. The structure and dynamics of tetrameric αSyn, taking into account factors that can influence the tetramer: monomer *ratio*, have been studied by NMR and computational studies on multimeric αSyn structure. Computational works explored the possibility that disordered monomers and metastable α-syn tetramers could coexist. An ensemble, containing mainly monomers (64.1% ± 6.4%), tetrameric species (28.2% ± 6%), and trimeric structures (7.7% ± 3.6%.), was constructed from replica exchange MD simulations together with a variational Bayesian weighting algorithm [57]. This algorithm defines a probability density of the population weights assigned to each structure from the trajectories. The simulations were performed with implicit solvent and using a CHARMM force field. The low-resolution starting models were obtained by fitting data from previous studies that suggested the presence of an amphipathic helix in the monomeric form to structures of other protein’s trimers and tetramers. Another model of the tetramer was derived from a limited set of NOEs from NMR at high concentrations (0.5 mM) of α-syn. The tetramers were found to be more abundant than trimers, and even though the initial structures were rich in helical content, a large fraction of them rearranged into strand-rich tetramers during the simulation.

Helix-rich tetramers can be found in physiological conditions and have been proposed as relevant to the α-syn homeostasis. To establish a relationship with the monomeric form and to understand what characteristics are important for the tetramer stabilization, Cote et al. applied MD simulations to a disordered monomer, a micelle-bound monomer and a helix-rich tetramer proposed by Wang et al. [56,58]. In these simulations, an analysis of local and global motions, together with an assessment of coarse-grained dihedral angles and steric coefficients, showed that the tertiary structure of the tetramers is particularly stable, and the helices present in the beginning of the simulations are little altered. This is explained by the hydrophobic interactions between the second helices (residues 54 to 83) of the four chains, which form a hydrophobic core, and by salt bridges between charged residues (lysines from the N-terminal domain, and glutamates and aspartates from the C-terminal domain). The analysis also showed that, with increasing temperature, the tetramer becomes increasingly disordered. The portions of the sequence that contain KT, KA and KK combinations, relevant for the formation of tetramers in physiological conditions, showed a low correlation between the side-chain and main-chain movements for the tetramer and the monomers, indicating a stabilizing role for these repeats.

### 2.3. Liquid-Liquid Phase Separation

In recent years, it has been demonstrated that α-syn could self-assemble into liquid droplets where the protein separates from the aqueous milieu, forming a new phase. This process is called liquid-liquid phase separation and has been shown to be a preliminary stage of the amyloid process that could occur before the formation of aggregates. In this new phase, α-syn stays in a liquid state showing a high dynamicity; however, these structures could mature into a gel-like state, rich in amyloids. As these structures have been observed not only in vitro but also in vivo, when cells are stressed, these droplets could be named as a membraneless compartment [59]. In the recent publication of Ray et al., the authors showed through fluorescence microscopy that, at high protein concentrations (200–500 μM), an α-syn forms droplets that can be induced in the presence of the molecular crowder PEG, inert polymers which significantly reduce the lag time for protofibril formation and the conversion of the protofibril into fibrils [60]. By fluorescence recovery after photobleaching (FRAP), they observed that these droplets are highly dynamic. Also, the analysis of the pathological mutants revealed that these latter undergo this phase transition, faster than WT protein. A deep structural analysis indicated that the major regions of the protein that contribute to the phase separation are the N terminus and hydrophobic NAC domain. These droplets then could form solid-like structures as hydrogels containing oligomers and fibrillar aggregates. These droplets were also observed in cells, suggesting that this could be a first stage of α-syn aggregation in the Lewy bodies [61]. Recently, Sawner et al. have studied in detail the factors that play a role in the formation of liquid droplets. Testing was carried out under different conditions, such as varying salt concentrations, pHs, presence of metals and bivalent cations, N-terminal acetylation, protein concentration, and time. It was shown that at physiological conditions, α-syn does not undergo phase separation, but the addition of compounds, e.g., bivalent cations or transition metals, and the high salt conditions could induce it. These results help in establishing the basis to evaluate α-syn phase separation [62].

It is worth noting that in the cellular context, α-syn could be in contact with other proteins and molecules that may affect or induce the phase separation or its partition. This was recently observed when α-syn was exposed to synaptic vesicle mimetics, which are highly abundant in the cellular localization of α-syn. In this sense, it was observed that these liposomes mimicking synaptic vesicles stabilize the liquid state of α-syn [21]; however, the incubation of this protein with negatively charged liposomes induces the progression to solid-like structures [60]. Furthermore, the presence of other proteins could affect the formation or partition of α-syn in liquid droplets; for instance, the full length α-syn partitions into tau/RNA droplets. By NMR spectroscopy it was revealed that the carboxy-terminal interacts with the proline-rich region of tau in tau droplets, suggesting that this protein partition into both phases could contribute synergistically in neurodegenerative pathologies [63]. Interestingly, it was recently observed that the liquid droplets produced by α-syn could be modulated by interaction with natural molecules. In particular, it has been observed that an antimicrobial peptide named as LL-III could not only interact with both α-syn monomers and condensates, but also stabilized the droplets and prevented their conversion into the fibrillar state [64].

## 3. Mechanisms of α-Syn Aggregation in Biological Systems

So far, we have focused on the recent advances in the description of the oligomerization mechanisms from an in vitro perspective. Understanding oligomerization in vivo is the basis for the study of the events that occur in living systems or in biological environments. The question that arises, then, is whether what we learn from in vitro assays can be applied in vivo. Recent advances in protein chemistry, biophysics, imaging, and proteomics have begun to be applied in research to decipher and embrace the complexity of the processes underlying protein aggregation, in a context where a fibril-centric approach has dominated the field over the past decades [65]. The system becomes even more complex if the inclusion formation, or rather the assessment of the types and distribution of non-proteinaceous components of amyloid plaques, is considered, as recently revealed by correlative light electron microscopy (CLEM) in LBs [66,67] and Huntingtin inclusions [68]. These works stress the urgent need for model systems that capture the entire process from protein misfolding to inclusion formation and maturation, and not only fibril formation.

A major issue which often happens while studying amyloidogenic proteins is the lack of adequate cellular models that enable the study of the aggregation process in living cells [69]. For example, in the case of α-syn, its low propensity to aggregation makes it difficult to mimic the process of formation of LB-like inclusions in cells. This prompted the researchers to find an artificial variant of α-syn, so-called SynT, to be used as a model of aggregation in mammalian cell systems. SynT is a modified form of α-syn displaying a higher propensity to aggregate and leads to the formation of inclusions which look like LBs upon co-expression with synphilin-1, an α-syn interacting protein [70,71]. The modification is on the C-terminus, where an extension from the proteolytic cleavage of enhanced green fluorescence protein (EGFP) is added. Since SynT has been proven to be a powerful and versatile system to model α-syn aggregation [72,73], a combination of complementary biophysical methods is considered to be a suitable approach to validate this model in terms of biochemical and biophysical properties in comparison with the wild-type variant. NMR studies combined with ThT fluorescence spectroscopy and TEM enabled demonstration that the conformations adopted by α-syn in its SynT form resemble those of the unmodified protein. Only some N-terminal alterations could be observed and these together with the hindrance of the C-terminus could be responsible for the decreased membrane SynT binding capacity, which might contribute to the aggregation behavior in a cell system [74]. In detail, intramolecular contacts between the C- and N- terminus are proposed to regulate the major events of nucleation [75,76,77]. Results from ensemble MD simulations with restraints obtained from paramagnetic relaxation enhancement (PRE) NMR spectroscopy show an ensemble with more compact species than those expected for a random coil due to interactions between the C-terminal domain and the fibril core region (NAC), reinforcing the idea that when this interaction is disturbed, aggregation is increased [75]. Moreover, long-range contacts determine an “open” conformation of the molecule that allows aggregation and alterations. Alterations of these long-range contacts promote exposures of the fibrillating segment of the NAC region enhancing the aggregation propensity [77,78] as observed also in vitro studies [13].

A recent neuronal model reproducing the key events which lead to the formation of LBs has been described in the literature [79]. By using integrative imaging approaches, this work provided a first example of the formation of organelle-rich LB-like inclusions in neurons, allowing the investigation of the molecular mechanism underpinning the seeding, fibrillization, and LBs formation in cells. It has been observed that the formation of α-syn fibrils occurs early, primarily in neurites, without significant alteration of the proteome. At this stage, a protein quality control machinery and other related processes are activated as cellular responses to prevent the formation or the accumulation of the fibrils in the cytosol. In a neuronal seeding model, major changes in the structural properties of the newly formed fibrils, occurring in a later stage, could be successively observed, together with a significant recruitment and aberrant sequestration of intracellular proteins related to the intracellular transport [79]. Compact α-syn is mostly localized in cell inclusions. Less compact α-syn is localized mostly in the filamentous neuritic fibrillar pathology.

**Figure 5:**
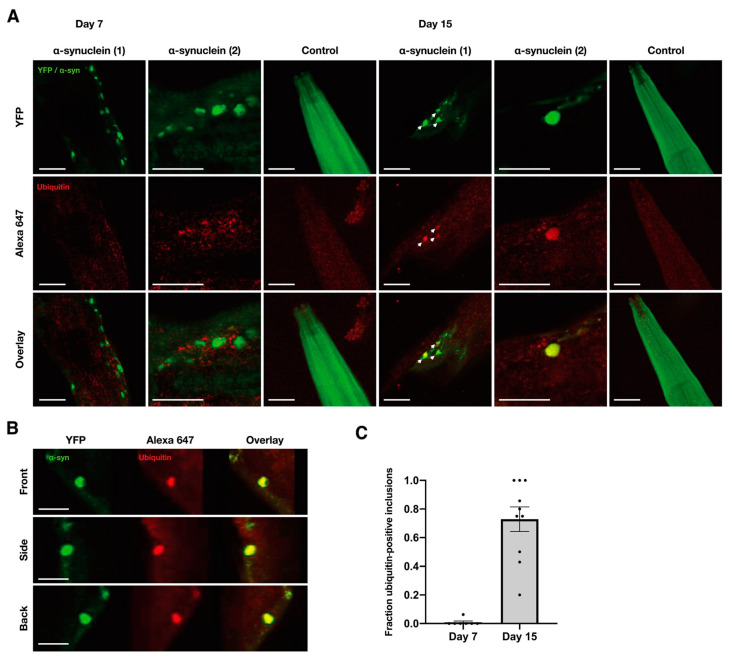
The study of α-synuclein inclusions in a model animal. (**A**) Inclusions in aged worms (Day 15, right) are immunoreactive for ubiquitin, whereas inclusions in younger worms (Day 7, left) do not show immunoreactivity. For each time point, two representative animals are shown together with a control strain expressing only YFP. Scale bar, 20 µm. (**B**) 3D rendering of an image stack showing co-localization of α-synuclein–YFP and Alexa 647 anti-ubiquitin signal in an aged worm (Day 15). Scale bar, 10 µm. (**C**) Quantification of ubiquitin-positive inclusions at indicated time points. Each data point represents the average number of ubiquitin-positive inclusions in one worm. Results are mean ± SEM. For further details refer to [21].

As mentioned before, α-syn might also form liquid droplets which then could progress to the formation of solid-state structures like fibrils. However, the formation of these structures in the cellular and animal model is complex and requires an extensive analysis. Recently, By FRAP analysis, it has been observed that cells overexpressing α-syn and extensively treated with iron presented liquid condensation in their cytoplasm, which decreased after 48 h. The diffusivity of the droplets was analyzed by single-particle tracking measurements inside cells, revealing that, after 24 h, these droplets display super-diffusive behavior which indicates that an active or facilitated motion of these droplets occurs inside the cell. On the other hand, α-syn is located in the nerve termination, an environment rich in proteins and vesicles and involved in the movement and fusion of neurotransmitters containing vesicles, thus making the analysis of this protein behavior in the presence of these molecules of great relevance. Recently, it has been described that, in a heterologous cell system, the protein synapsin, a synaptic protein, generates condensates where α-syn can be recruited and maintain high mobility [80]. Moreover, a study on Caenorhabditis elegans PD as an α-syn animal model has shown that α-syn forms liquid droplets, followed by their conversion into an amyloid-rich hydrogel with Lewy-body-like properties, having characteristic post-translational modifications such as ubiquitination. These results suggest that liquid droplet condensate arrestation in vivo may be linked to the maturation and formation of Lewy bodies in a cellular context (Figure 5) [21].

## 4. Conclusions and Perspectives

In summary, we explored some selected examples of complementary techniques and approaches that were recently applied to study the early stages of α-synuclein oligomerization and fibrillation in vitro and in vivo. The complexity and diversity of α-syn aggregate species are common features to all other amyloid proteins. Generally, amyloid proteins are largely unstructured in solution in their monomeric state and can be described as natively unfolded or intrinsically disordered, except when they fold in a specific conformation after interaction with a binding partner. In some circumstances, these proteins can convert into nonfunctional and toxic aggregates, often because they adopt a conformation favoring their aggregation propensity. In this review, it can be noticed that α-syn can give rise to highly disordered, partially structured, or native-like oligomers, depending on whether they originated from unfolded, partially folded, or folded monomeric states, respectively. α-syn seems to undergo a transition from unstructured conformations into β-sheet-rich fibrils passing through helix-rich intermediates with the involvement of the N-terminal and central portions in the formation of helix structures, and the mainly extended conformation of the C-terminus. Dimer formation might be the early event responsible for the onset of aggregation. Helix-rich dimers seem to be more flexible, with continuous unfolding transitions, compared to the ones characterized by a high content of β-sheets, and prone to establish intermolecular edge-to-edge interactions stabilizing the dimer nucleus. Hydrophobic interactions in the NAC region seem to be key to dimer formation. Weak intermolecular interactions characterize the early aggregates, which are rather unstable and can dissociate to generate other types of soluble oligomeric species. These initial oligomers can be either a cluster that promotes further self-association of monomers, thus generating larger aggregates that further grow into fibers, or a simple nucleated seed which undergoes internal structural reorganization towards species with increased compactness and size. Secondary structure and surface-exposed hydrophobicity play an interconnected role in aggregate toxicity, with the ones with lower parallel β-sheet content and higher hydrophobic area being the most toxic. Together with an on-pathway process forming fibrils, there is an off-pathway mechanism, which can either generate oligomers responsible for the toxicity or large amorphous deposits. These oligomers are often disordered-aggregates or native-like aggregates which can grow without substantial conversion of their secondary and tertiary conformation. By using different complementary analyses, it was possible to characterize type-A oligomers as precursors not having a defined structure and most probably related to Meta-αS-Os. These structures can be converted into more compact type-B oligomers with amyloid-like β-sheet conformation, which participate in fibril production. Among type-B oligomers, it is possible to distinguish between two types of oligomers: type-B*_on_* which play the most important role in the fibrillation process, and type-B*_off_* which are instead characterized by a high level of antiparallel β-sheet content and whose transformation into parallel sheets is energetically unfavorable, so that they no longer participate in the fibril formation. By FRET analysis, instead, two distinct subtypes of α-syn oligomers have been identified: species with high and low FRET. α-syn aggregation might be characterized by a first transition of the initially formed oligomers (low FRET, globular) to more compact oligomers (high FRET) which successively proceed to fibril formation by monomer addition. More recently, it has been shown that, during the early stages of aggregation, oligomer-oligomer interactions seem to drive the fibrillation more so than other type of interactions, such as those between oligomers and monomers, suggesting that early-stages oligomers do not behave as seeds by the addition of monomers.

As stated above, α-syn aggregation clearly appears as a complex process characterized by multiple pathways, which obviously increases the complexity of its evaluation. Moreover, these multiple pathways sometimes depend on experimental conditions and on the conformational state adopted by the monomer in these conditions. Electron microscopy (EM) and atomic force microscopy (AFM) techniques allow us to characterize the morphology, size and compactness of the aggregates, while Fourier transform infrared spectroscopy, solid-state nuclear magnetic resonance (ssNMR), and X-Ray crystallography can go deeper inside the conformation adopted by the amyloid protein into aggregates. Bulky techniques such as fluorescence spectroscopy by employing binding dyes (i.e., Thioflavin T) or Circular Dichroism monitor the existence of such structures overtime. By reviewing recent research studies on α-syn aggregation, we showed that the application of robust experimental and mathematical analyses is a fruitful approach to analyze the kinetics of amyloid formation and to yield insight into its mechanism. In summary, it is explicit that making interconnections between morphology, topology and kinetics is decisive for furnishing a comprehensive portrait of the mechanism of amyloid aggregation in vitro and in biologically relevant systems.
